# Repeated successful use of eltrombopag in chronic primary immune thrombocytopenia: description of an intriguing case

**DOI:** 10.1002/ccr3.920

**Published:** 2017-07-13

**Authors:** Cristina Santoro, Paola Volpicelli, Erminia Baldacci, Grazia Ferrara, Alice Di Rocco, Antonietta Ferretti, Marika Porrazzo, Maria Gabriella Mazzucconi

**Affiliations:** ^1^ Hematology Department of Cellular Biotechnology and Hematology “Sapienza” University of Rome Italy

**Keywords:** Eltrombopag, ITP, therapy, TPO‐RA

## Abstract

Thrombopoietin receptor agonists (TPO‐RAs) are used as effective alternative treatments in ITP patients unresponsive to first‐/second‐line therapies. TPO‐RAs can also be used to normalize platelet count to safely perform invasive procedures and chemotherapy, in case of malignancies. In few responsive patients, TPO‐RAs can be suspended maintaining a sustained response.

## Introduction

Primary immune thrombocytopenia (pITP) is an autoimmune disorder characterized either by an increased peripheral platelets clearance or by a decreased platelets production. The goal of pITP therapy is to reach and maintain a safe platelet level to avoid bleedings. International guidelines recommend corticosteroids and/or high‐dose immunoglobulin as first‐line treatment, while splenectomy as a second‐line therapy. In case of contraindication or refractoriness to splenectomy, rituximab or other immunosuppressants and the new thrombopoietin receptor agonists (TPO‐RAs), romiplostim and eltrombopag, are considered alternative treatments 1, 2. In Europe, TPO‐RAs have been approved by EMA for the treatment of chronic pITP patients unresponsive to other therapies.

## Case Report with Results

In May 2007, a 61‐year‐old woman was referred to our center because of a severe thrombocytopenia (platelet count 17 × 10^9^/L) and presence of ecchymoses and petechiae. Bone marrow smear examination showed increased megakaryopoiesis and normal granulopoiesis, erythropoiesis, and lymphopoiesis. Other causes of ITP (autoimmune conditions, infections, solid or hematologic neoplasms) were excluded. Therefore, a diagnosis of pITP was made and the patient started corticosteroid therapy (prednisone 1 mg/kg/day for 4 weeks) obtaining a complete response (CR) with a platelet count of 160 × 10^9^/L. She could then taper prednisone and stop it after 3 months since start. During follow‐up, the patient relapsed three times (February 2009, August 2010, and September 2011). During each episode, platelet count was <30 × 10^9^/L with the presence of cutaneous bleedings; the patient was treated again with prednisone, 0.5–1 mg/kg/day, obtaining a transient CR, after the first and the second episode, while after the third one, corticosteroid therapy was ineffective. Due to the age of the patient (65 years) and her poor compliance, splenectomy was considered contraindicated. Moreover, we did not consider rituximab and other immunosuppressants convenient for an old patient who was just given corticosteroids. Consequently, in December 2011, treatment with eltrombopag was started at a dosage of 50 mg/day, then, increased up to the maximum dosage of 75 mg/day. At the seventh week of treatment, eltrombopag was suspended because of high platelet count (650 × 10^9^/L). A week later, platelet count normalized (210 × 10^9^/L). Subsequently, the patient obtained a sustained CR off therapy, without other treatments or rescue therapies, with a platelet count ranging between 115 and 320 × 10^9^/L (median 156 × 10^9^/L). In July 2013, the patient came back to our center, because of altered values of blood tests. In particular, she presented microcytic anemia (Hb 8.9 gr/dL, MCV 75 fL), normal number and differential count of white blood cells, normal platelet level (118 × 10^9^/L), increased inflammation indexes (erythrocyte sedimentation rate 60, C reactive protein 6 mg/dL), and polyclonal hypergammaglobulinemia. Neither iron deficiency nor autoimmune hemolytic anemia were found. Liver and spleen enlargement observed at the physical exam was confirmed by a CT scan, which also showed subdiaphragmatic lymphoadenopathies (short‐ and long‐axis diameters <2 cm). Serologic evaluation for HIV, HCV, HBV infections was negative. A bone marrow examination did not show abnormalities; peripheral blood immunophenotype evaluation did not provide evidence of a monoclonal B‐ or T‐lymphocyte population; moreover, the patient was negative for JAK2 mutation. A bone marrow biopsy did not show either fibrosis, or a lymphoproliferative disorder. Therefore, at that time we could not formulate a diagnosis. In October 2013, after 20 months of sustained CR, the patient relapsed (platelet count 4 × 10^9^/L). Because of the previous response, eltrombopag was restarted (50 mg/day), without concomitant corticosteroids administration. After 2 months of treatment, the patient obtained again a CR (platelet count 164 × 10^9^/L). In January 2014, eltrombopag was suspended and the patient reached a second sustained CR off treatment. In the mean time, a further bone marrow immunophenotype evaluation documented a clonal B‐lymphocytic population (CD20/CD38+, CD103−, IgG‐lambda restricted) and a bone marrow biopsy showed poor infiltration of large sized atypical lymphoid cells in the context of a large number of reactive T lymphocytes. At immunohistochemistry evaluation, the atypical lymphoid cells were CD20 positive; BCL6 and CD10 were not evaluable. The evidence of these atypical large B cells allowed a diagnosis of aggressive B‐cell lymphoma of probable splenic origin. Then, the patient underwent six cycles of immune‐chemotherapy with rituximab, cyclophosphamide, vincristine, prednisone (R‐CVP) every 3 weeks from February to July 2014. At the end of the sixth cycle, she obtained a complete remission according to Cheson criteria: negative CT/PET‐scan and bone marrow biopsy 3. During the whole period of immune‐chemotherapy, the patient never relapsed for ITP. In November 2015, a CT scan performed for the presence of B symptoms (fever, joint pain, and night sweats) documented a relapse of lymphoma (splenomegaly and diffuse lymphoadenopathies). For this reason, the patient underwent immune‐chemotherapy with rituximab and bendamustine for six planned courses. At the most recent control (May 2016), she is in sustained CR for ITP, which is persisting after 26 months (platelet count 121 × 10^9^/L). We cannot completely exclude that treatment with corticosteroids and rituximab which are part of the lymphoma therapy could help to maintain the ITP sustained response. Partial remission of the lymphoma has been documented by a CT scan.

## Discussion

In this case report, eltrombopag was used as second‐line therapy for loss of response to corticosteroids. The patient was treated for two short periods, obtaining a CR and maintaining a sustained CR off therapy, lasting 20 and 26 months, respectively. In the last few years, literature reports have shown the possibility of maintaining response after discontinuation of TPO‐RAs, in up to 10–40% of patients treated either with romiplostim or eltrombopag 4, 5, 6, 7, 8, 9, 10. In particular, as regard as eltrombopag, Leven et al. 4 described a case series in which 5/15 (33%) patients could stop eltrombopag and maintained a sustained CR for ≥5 months off treatment. Gonzalez‐Lopez et al. 8 reported on the successful discontinuation of eltrombopag on a pITP case series of 260 patients: 201 obtained a CR and the rate of sustained response off therapy was 10% (26/260). As regard as pathophysiological mechanisms underlying this phenomenon, Ghadaki et al. 5 suggested that TPO‐RAs, increasing the exposure of immune system to platelet antigens, may restore immune tolerance to platelets, as similarly it is hypothesized for immune tolerance induction in patients with hemophilia and inhibitory alloantibodies to FVIII. Moreover, the TPO‐RAs themselves may have a direct effect on restoring normal T‐regulatory function 11.

Our patient relapsed after 20 months of sustained CR. She was treated again with eltrombopag obtaining a second sustained CR lasting 26 months. Therefore, she could safely perform invasive procedures as bone marrow biopsy and undergo immune‐chemotherapy. Our experience is in agreement with results of the “REPEAT” study from Bussell et al. 12. In this study, the authors explored the cyclic use of eltrombopag (50 mg/day for 6 weeks, then 4 weeks off treatment for three cycles) demonstrating that loss of response occurred between cycles could be recovered in the most part of patients (87% of responders to the first cycle) using the same TPO‐RA. Data are also reported about the use of TPO‐RAs for the treatment of thrombocytopenia related to hematologic, autoimmune, and infectious diseases, or to chemotherapy 13, 14, 15, 16, 17, 18, 19. In particular, the use of eltrombopag in chronic lymphocytic leukemia (CLL) associated ITP was described by different authors 13, 14, 15: in two cases, a CR was obtained and one patient could stop the drug 13; in one case, eltrombopag was successfully used preoperatively to increase the platelet count to allow laparoscopic splenectomy 14; in one other case, eltrombopag increased platelet count to normal range 13 days after treatment start in a patient who presented recurrent ITP associated to pulmonary hemorrhage 15. Al‐Nawakil et al. 16 reported the use of romiplostim in four ITP patients during the diagnostic phase or after diagnosis of lymphomas: 3/4 patients obtained a response. Gudbrandsdottir et al. 17 reported the use of both TPO‐RAs in a series of patients affected by either pITP or by other disorders; thirty‐two cases were included in this study: 15 received TPO‐RAs for pITP, 7 for secondary ITP (CLL, LES, Evans syndrome, HIV infection, and celiac disease), 10 for nonimmune thrombocytopenia (chemotherapy, acute myeloid leukemia, myelodysplastic syndrome, hereditary spherocytosis, and suspected chemically induced thrombocytopenia). Response was obtained in 59% of pITP patients, in 57% of secondary ITP, and in 40% of patients with nonimmune thrombocytopenia. The use of TPO‐RAs in lymphoproliferative disorders has been reported even in one randomized study by Moskowitz et al. 18: a first generation TPO‐RA (PEG‐MGDF) was randomly introduced in the chemotherapeutic regimen ICE for treatment of 41 patients affected by refractory/relapsed diffuse large‐cell lymphomas (DLCL). This therapy could improve platelet count in patients with thrombocytopenia treatment related and consequently allowed to keep dose‐intensity chemotherapy. Demeter et al. 19 described a case of a patient, belonging to Yehova's witnesses, affected by mantle cell lymphoma, treated with intensive chemotherapy (Hyper CVAD): on day 13 from the start of chemotherapy, a severe thrombocytopenia developed associated with gastrointestinal bleeding, but the patient did not accept platelet transfusions. A single dose of 1000 mcg of romiplostim was given obtaining a quick normalization of the platelet count. In all these reports, the authors highlighted the benefits of TPO‐RAs use, either for their efficacy on increasing platelet count to perform invasive procedures and chemotherapy or for their safety profile in patients who are severely immunosuppressed because of the underlying disease or concomitant therapies. TPO‐RAs are efficacious in the treatment of pITP, and in some cases, it is possible to reach a sustained response after discontinuation. Moreover, in case of relapse, treatment with the previously used TPO‐RA could induce a further response. TPO‐RAs can be used not only for pITP, but also in case of thrombocytopenia related to other disorders or chemotherapy. In this subset of patients, the response rate is high, confirming the efficacy of TPO‐RAs also in secondary immune or nonimmune thrombocytopenia.

## Authorship

MGM, CS, PV: wrote the manuscript. EB and ADR: performed clinical management of the case. AF, MP: helped in clinical management of the case. GF: carried out the literature search and revised the manuscript.

## Conflicts of Interest

MGM: Speaker's bureau: Amgen, Baxter, Bayer, Kedrion, Novartis, Novonordisk, Pfizer, Shire. Advisory board: Baxter, Novonordisk. CS: Speaker's bureau: Baxter, Kedrion, Novartis. Advisory board: Baxter, Bayer, Pfizer, SOBI. EB: Speaker's bureau: Amgen, Bayer, BIOVIIIX, Pfizer. ADR: Speaker's bureau: Celgene, Janssen, Roche. Advisory board: Amgen. PV, GF, AF, and MP: declare no conflict of interests.

## Ethics Statement

We report a case of chronic ITP treated with eltrombopag and perform a literature review. No experiments were performed on human subjects. Patient anonymity was maintained throughout the manuscript.
